# A multilevel mixed-effects regression analysis of the association between hospital, community and state regulatory factors, and family income eligibility limits for free and discounted care among U.S. not-for-profit, 501(c)(3), hospitals, 2010 to 2017

**DOI:** 10.1186/s12913-021-06219-4

**Published:** 2021-03-15

**Authors:** Jason N. Mose

**Affiliations:** 1 Department of Health Services and Information Management, East Carolina University, Greenville, North Carolina USA; 2 School of Nursing, University of North Carolina at Chapel Hill, Chapel Hill, USA

**Keywords:** Tax-exempt hospitals, Charity care, Free care, Discounted care, Community benefits, Schedule H, Form 990

## Abstract

**Background:**

Not-for-profit hospitals are facing an uncertain financial future, especially following the COVID-19 pandemic. Nevertheless, they are legally obligated to provide free and discounted health care services to communities. This study investigates the hospital, community, and state regulatory factors and whether these factors are associated with family income eligibility levels for free and discounted care.

**Methods:**

Data were sourced from Internal Revenue Service Form 990, several data files from the Centers for Medicare and Medicaid, demographic and community factors from the Census Bureau, supplemental files from The Hilltop Institute, Community Benefit Insight, and Kaiser Family Foundation. The study employs multilevel mixed-effects linear and ordered logit regressions to estimate the association between the hospital, community, state policies, and the hospital’s family income eligibility limit for free and discounted care.

**Results:**

A plurality of hospitals (49.96%) offered a medium level of family income eligibility limit (160–200% of the federal poverty level (FPL)) for free care. In comparison, about 53% (52.94%) offered a low level (0–300 of FPL) eligibility limit for discounted care. Holding all else equal, hospitals designated as critical access, safety net, those in rural areas or located in disadvantaged areas were associated with an increased probability of offering low eligibility limits for free and discounted care. Hospitals in a joint venture, located in highly concentrated markets or states with minimum community benefits requirements, were associated with an increased probability of offering high eligibility limits.

**Conclusion:**

State and community factors appear to be associated with the eligibility level for free and discounted care. Hospitals serving low-income or rural communities seem to offer the least relief. The federal and state policymakers might need to consider relief to these hospitals with a requirement for them to provide a specific set of minimum community benefits.

## Background

The Patient Protection and Affordable Care Act (ACA), enacted in 2010, included additional requirements for private not-for-profit (NFP) and some government-owned hospitals that are regulated under section §501(c)(3) of the Internal Revenue Code. For example, applicable hospitals must have a written financial assistance policy (FAP), which stipulates, among other things, “eligibility criteria for financial assistance and whether such assistance includes free or discounted care” [1]. In 2014, the Internal Revenue Service (IRS) published a final rule implementing the ACA provisions. The final regulations further require the FAP to apply to all emergency and other medically necessary care provided by the hospital. It must be widely publicized and must include the eligibility criteria for financial assistance as required under ACA [2]. One of the eligibility criteria widely used to qualify for free or discounted care is family income based on the federal poverty levels (FPL). Given the heterogeneity of hospitals, communities, and state policies, it is unknown whether there are variations on the FPL levels. Hospitals apply for free and discounted care based on hospital community characteristics. Further, little is known to what extent state regulatory policies influence the limits for which hospitals can apply and whether the eligibility limits have changed since the enactment of ACA in 2010.

Several factors and developments make eligibility limits for free and discounted care salient for families and timely for policymakers to reconsider. First, the NFP comprises 69% of non-government community hospitals [3], and therefore accessibility to free and discounted care may be a lifeline for low-income families, the uninsured, or underinsured individuals. Findings from surveys indicate that the uninsured are from low-income families, have a working member in the family, and are more likely to be adults than children [4]. The number of uninsured first dropped from 46.5 million in 2010 to 27 million in 2016 [4]. However, the number of uninsured has increased in subsequent years. Further, the number of those purchasing their coverage through the marketplace has continued to trend downward since 2016 [4, 5]. The COVID-19 pandemic has exacerbated the uninsured trend, with more people potentially losing their insurance coverage as the country goes through economic uncertainty, and more employers struggle financially [6, 7].

Second,, individuals and families who fall in the insurance coverage and assistance gap (those who do not qualify for Medicaid and ACA insurance subsidies) in Medicaid non-expansion states might also face challenges in accessing care. Research indicates that those in the gap tend to have lower levels of education, have a chronic condition, have dependents that make them more vulnerable and need financial assistance [8]. Further, the combination of state decision policies such as expanding Medicaid, Medicaid eligibility limits, and overall health care cost can have a negative effect on individuals seeking care and, in some cases, exacerbate health disparities [9, 10].

The uninsured or underinsured would likely turn to the local NFP hospitals for free or discounted care. The same NFP hospitals, which traditionally have a thin operating margin, will be struggling themselves following the loss of volume and, as a result, revenue during the lockdown and disruptions [11–14]. Despite the recent appropriations from Congress, the grants are estimated to cover about a month’s worth of lost income, and the hospitals will continue to face financial uncertainty [11]. Some of the NFP hospitals were already facing financial headwinds following the uptick of uninsured rate, scrutiny for surprise billing, and stricter Medicaid eligibility requirements in some states [15]. As states cut Medicaid due to the pandemic, NFP hospitals will be left to grapple with their legal obligation to provide community benefits while staying afloat [16].

The third category of people who might rely on free or discounted care are families that face a medical emergency, sudden illness, and have expensive chronic conditions [17]. Even with adequate insurance coverage, some treatments are expensive for families to afford, and as a result, require financial assistance through discounts. Also, more individuals and families are being enrolled, mostly through their employer, on high deductible plans [18]. Added to this, the phenomenon of surprise billing and aggressive collection policies by some NFP hospitals often leads to desperation in families to qualify for free or discounted care [19–22].

This study seeks to, first, investigate whether hospital characteristics and community factors are associated with the family income eligibility limits for free and discounted care. The study hypothesizes that large, not-for-profit churches, major teaching and system hospitals would be likely to set over-generous limits for both free and discounted care. The idea is that these hospitals are likely to have multiple sources of revenue that will enable them to subside the free and discounted care. Conversely, safety-net, critical access, rural and those located in most disadvantaged areas will be less likely to offer more generous eligibility limits. Second, estimate the influence, if any, of state laws or regulatory policies on eligibility limits. The study hypothesizes that hospitals in states with conditional community benefit requirement, stricter community benefit plan than the federal requirement, and minimum community benefit requirements, on average, will have higher eligibility limits for free and discounted care.

## Methods

This study’s data was sourced from IRS, Centers for Medicare and Medicaid Services (CMS) data, U.S. Census Bureau, and other supplemental datasets from The Hilltop Institute, and Community Benefit Insight. Data for the tax year 2010 to 2017 were extracted from IRS Form 990, Schedule H data. Two continuous and two categorical dependent variables were created for family income eligibility limit for free and discounted care based on federal poverty level guidelines. The categorical dependent variables were divided into three quantiles and categorized as low, medium, or high eligibility levels. The low category for free care ranged from 40 to 150% of the FPL, the medium category ranged from 160 to 200% and the high category ranged from 201 to 600% of the FPL. For the discounted care, the low category ranged from 0 to 300% of PFL, medium-ranged from 301 to 400%, and high category ranged from 401 to 800% of PFL. The dependent variable was selected because it is widely used for eligibility purposes and yet little is known whether the choice of the cut points is associated with hospital service area or state factors.

Hospital factors such as teaching status, safety-net status, hospital size, hospital ownership, hospital system status, and market concentration among other variables, were collected from CMS’ provider of service (POS), impact, and hospital service area file. Teaching status was defined as major, minor and non-teaching as reported under medical school affiliation in the POS file. Safety-net hospitals were defined as those that fall in the top quartile of Medicare Disproportionate Share Hospital (DSH) patient percent. Hospital size was categorized as small (0–99 beds), medium (100–399 beds) and large (400+ beds). Hospital ownership was reported in three categories: not-for-profit private (reference), not-for-profit church and public (government owned). A hospital also was categorized as a system hospital if it was reported in the POS as a multi-hospital facility. Herfindahl–Hirschman Index (HHI), a measure of hospital concentration, was calculated for each year using total inpatient days. Further, the hospitals’ markets were broken into three quantiles with the highest quantile categorized as highly concentrated, the second as moderately concentrated, and the lowest quantiles as lowly concentrated.

Community factors, which were at the hospital service area or county level, such as racial composition, and seventeen variables used to create area deprivation index, an indicator for area disadvantage, were sourced from the American Community Survey (ranging from 2007 to 2011 to 2014–2018) [23]. The area disadvantage was created at the county level, and further divided into four categories. The most disadvantaged was the counties that fell in the top 5% in the area deprivation index. The second and third disadvantaged were those counties in the second and third top 5% in the deprivation index. The lower 85% counties on the deprivation index were used as the reference category. State policy variables (such as minimum community benefits requirement, sales tax exemption, conditional community benefits requirement) were sourced from The Hilltop Institute at the University of Maryland Baltimore County [24]. Other variables were extracted from the Community Benefit Insight tool, maintained by the Research Triangle Institute [25].

The study employs multilevel mixed-effects linear regressions in a panel data analysis to estimate the association between the hospital, community, state policies, and the hospital’s family income eligibility limit for free and discounted care. To estimate the probability of having a low, medium, or high family income eligibility limit, the study fitted multilevel mixed-effects ordered logit regression. In both cases, the multilevel regression analysis approach was chosen because of the data’s nested nature; a hospital is nested in the service area and is nested in the hospital referral region. The study posits that hospitals in a competitive market might offer a higher free and discounted family income limit than a less competitive service area or referral region. In other words, a hospital might be influenced by their peer hospital in the same service or referral region more than the national trend or eligibility limit.

Further, the multilevel models allowed simultaneously estimation of possible effects of hospital characteristics, community factors, and state policies that might influence free and discounted care eligibility while producing correct standard errors for community and state-level predictors [26]. The Intraclass Correlation Coefficient, after the mixed linear regressions, revealed that 61.41% (95% CI, 53.23–69%) and 60.51% (95% CI 55.19–65.60%) of free and discounted care eligibility limits can be explained by hospital service areas nested in the hospital referral regions. For the multilevel models, we followed the steps suggested by Sommet and Morselli [27]. Based on the recommendation, the study question, and documented best practices; first, the data was prepared by grand-mean centering the first level (hospital) variables [26, 28]. Next, an empty model was built, and an intraclass correlation coefficient was calculated. A final model was built by testing if the inclusion of some specific variable and high ordered terms improved the models’ fit. Then testing was done to check whether the inclusion of random slopes of some variables improves the fit.

The analyses were conducted using Stata 16.1.

## Results

The number of individual hospitals ranged from 2021 to 2244 each year, totaling 16,269 and 16,378 hospital years for free and discounted care analyses. The average FPL limit for free care was 186 (with a minimum of 40 to 600) and 324 for discounted care (with a minimum of 0 and maximum of 800). Table 1 shows the proportion of hospitals by key variables, with hospital service area level adjusted standard errors and logistic transformed confidence intervals. On the one hand, a plurality of hospitals, 49.96% [95 CI 47.87–52.05%], provided a medium level (160–200% of the FPL) for free care eligibility limit. On the other hand, 52.94 [95 CI 50.85–55.02%] belonged to the low category (0–300% of FPL) in terms of discounted care family income eligibility limits. Concerning the state policies, about 39% were in a state that required the hospital to provide community benefit as a condition of the hospital’s exempt status. Also, 15.01 [95 CI 12.66–17.72%] were in states that required minimum community benefit, and 22.33 [95 CI 19.92–24.94%] were in a state that required a stricter community benefits plan than what IRS requires. Finally, Table 1 also shows that 14.34 [95 CI 12.49–16.41%] of the hospitals were in a state that does not exempt the NFP hospitals from sales or use taxes.

**Table 1:** Proportion of not-for-profit hospitals by hospital, community, and state regulatory factors

	Category	N	Percent	Robust Std. Err.	Logit [95% Conf. Interval]	
Family income eligibility limit category for free care	Low (40–150% of FPL)	5585	34.39	1.05	32.36	36.47
	Medium (160–200% of FPL)	8113	49.96	1.06	47.87	52.05
	High (201–600% of FPL)	2541	15.65	0.71	14.31	17.09
Family income eligibility limit category for discounted care	Low (0–300% of FPL)	8671	52.94	1.06	50.85	55.02
	Medium (301–400% of FPL)	5988	36.56	0.97	34.67	38.48
	High (401–800% of FPL)	1721	10.51	0.77	9.09	12.12
Critical access hospital	No	11,935	72.87	1.09	70.68	74.95
	Yes	4443	27.13	1.09	25.05	29.32
Safety-net	No	14,157	86.44	1.01	84.34	88.3
	Yes	2221	13.56	1.01	11.7	15.66
Public	No	15,168	92.61	0.51	91.55	93.54
	Yes	1210	7.39	0.51	6.46	8.45
Not-for-profit, church	No	14,516	88.63	0.63	87.34	89.8
	Yes	1862	11.37	0.63	10.2	12.66
Small hospital (< 100 beds)	No	8638	52.74	1.2	50.38	55.09
	Yes	7740	47.26	1.2	44.91	49.62
Medium (100–399 beds)	No	9714	59.31	1.03	57.26	61.31
	Yes	6664	40.69	1.03	38.69	42.74
Large (400+ beds)	No	14,404	87.95	0.79	86.31	89.42
	Yes	1974	12.05	0.79	10.58	13.69
Major teaching	No	14,467	88.33	0.9	86.44	89.98
	Yes	1911	11.67	0.9	10.02	13.56
Minor teaching	No	13,794	84.22	0.73	82.73	85.6
	Yes	2584	15.78	0.73	14.4	17.27
In a joint venture	No	10,454	63.83	0.92	62	65.62
	Yes	5924	36.17	0.92	34.38	38
Health system hospital	No	4134	25.24	0.93	23.46	27.11
	Yes	12,244	74.76	0.93	72.89	76.54
Moderately concentrated (0.3387 to 0.8401 on HHI)	No	11,121	67.9	1.13	65.64	70.08
	Yes	5257	32.1	1.13	29.92	34.36
High concentrated (0.8403 to 1 on HHI)	No	11,126	67.93	1.12	65.69	70.08
	Yes	5252	32.07	1.12	29.92	34.31
Rural (non-core)	No	12,716	77.64	1.01	75.61	79.55
	Yes	3662	22.36	1.01	20.45	24.39
Most disadvantaged	No	16,236	99.13	0.16	98.75	99.4
	Yes	142	0.87	0.16	0.6	1.25
Second most disadvantaged	No	16,113	98.38	0.21	97.92	98.74
	Yes	265	1.62	0.21	1.26	2.08
Third most disadvantaged	No	16,059	98.05	0.21	97.58	98.42
	Yes	319	1.95	0.21	1.58	2.42
Conditional community benefit requirement	No	9963	60.83	1.55	57.76	63.81
	Yes	6415	39.17	1.55	36.19	42.24
Minimum community benefit requirement	No	13,920	84.99	1.29	82.28	87.34
	Yes	2458	15.01	1.29	12.66	17.72
Stricter community benefit plan	No	12,721	77.67	1.28	75.06	80.08
	Yes	3657	22.33	1.28	19.92	24.94
No sales/use tax exemption	No	14,029	85.66	1	83.59	87.51
	Yes	2349	14.34	1	12.49	16.41
Medicaid covers non-disabled adults	No	8846	54.01	1.05	51.95	56.06
	Yes	7532	45.99	1.05	43.94	48.05
West	No	13,967	85.28	1.02	83.17	87.16
	Yes	2411	14.72	1.02	12.84	16.83
Northeast	No	12,966	79.17	1.28	76.56	81.56
	Yes	3412	20.83	1.28	18.44	23.44
Midwest	No	10,672	65.16	1.48	62.2	68.01
	Yes	5706	34.84	1.48	31.99	37.8
South	No	11,528	70.39	1.45	67.47	73.16
	Yes	4850	29.61	1.45	26.84	32.53

Table 2 presents multilevel linear mixed-effects analysis results of family income eligibility limits for free and discounted care. In both cases, free and discounted care, the first model is reduced from including only hospital and year effects factors, while the second is a full model including hospital, community, state policies, and years effects variables. Overall, the results indicate that the magnitude was smaller for those statistically significant variables in the reduced form once community and state regulatory factors were included. Generally, in terms of free care eligibility, critical care and safety-net hospitals are associated with 9.086 (*p* < 0.05) and 14.013 (*p* < 0.01) percentage points lower than non-critical care and non-safety-net hospitals, respectively, holding all else equal. For discounted care, critical access hospitals were associated with a 19.352 (*p* < 0.01) percent lower eligibility limit than non-critical care hospitals. Further, on the one hand, several factors were associated with lower eligibility limits. For example, a hospital located in a rural area (17.05%, *p* < 0.01), second-most disadvantaged area (9.22%, *p* < 0.01), and is located in a state with a conditional community benefit requirement (15.74%, *p* < 0.01), was associated with a statistically significant lower family income eligibility limit for free care. Similarly, concerning discounted care, a rural hospital (13.39%, *p* < 0.05) located in the most disadvantaged area (34.22%, *p* < 0.01) was associated with statistically lower eligibility limits.

**Table 2:** Family income eligibility limits for free and discounted care multilevel linear mixed-effects analysis results

	Family income eligibility limits for free care (as a % of FPL)		Family income eligibility limits for discounted care (as a % of FPL)	
	Hospital & year factors	Full model	Hospital & year factors	Full model
Non-critical access hospital	ref	ref	ref	ref
Critical access hospital	−15.316^***^ (− 3.453)	−9.086^**^ (−2.129)	− 24.727^***^ (− 3.660)	−19.352^***^ (− 2.965)
Non-safety-net	ref	ref	ref	ref
Safety-net	−14.081^***^ (− 3.625)	−14.013^***^ (− 3.618)	−8.396 (− 1.175)	−8.233 (− 1.149)
Not-profit-private	ref	ref	ref	ref
Public	−4.966 (− 1.071)	−5.171 (− 1.121)	−13.792^*^ (− 1.647)	−13.161 (− 1.572)
Not-for-profit, church	5.229 (1.373)	5.006 (1.320)	1.385 (0.205)	1.423 (0.210)
Small (< 100 beds)	ref	ref	ref	ref
Medium (100–399 beds)	0.174 (0.037)	−0.718 (− 0.152)	11.009^*^ (1.909)	10.320^*^ (1.797)
Large (400+ beds)	−4.506 (−0.584)	− 4.929 (− 0.642)	12.901 (1.495)	12.672 (1.464)
Non-teaching	ref	ref	ref	ref
Major teaching	9.194^**^ (1.962)	9.102^*^ (1.944)	9.497 (1.571)	9.565 (1.583)
Minor teaching	−1.408 (− 0.418)	−1.626 (− 0.488)	0.446 (0.072)	0.197 (0.032)
Not in a joint venture	ref	ref	ref	ref
In a joint venture	3.905 (1.114)	3.668 (1.050)	15.988^***^ (3.784)	15.663^***^ (3.707)
Stand-alone hospital	ref	ref	ref	ref
Health system hospital	5.239 (0.939)	5.198 (0.943)	4.568 (0.652)	4.571 (0.656)
Low concentrated (< 0.33 on HHI)	ref	ref	ref	ref
Moderately concentrated (0.3387 to 0.8401 on HHI)	−0.170 (− 0.078)	−1.401 (− 0.641)	4.746 (1.475)	4.044 (1.268)
High concentrated (0.8403 to 1 on HHI)	8.343^**^ (2.388)	5.921^*^ (1.704)	15.594^***^ (2.751)	14.145^**^ (2.538)
Financial assistance share of total expense	0.728 (1.165)	0.749 (1.189)	2.700^***^ (3.628)	2.584^***^ (3.406)
Northeast	−6.330 (− 0.808)	5.304 (0.754)	−28.126^**^ (− 2.433)	−34.512^**^ (− 2.554)
Midwest	− 19.343^**^ (− 2.250)	−6.755 (− 0.970)	−43.843^***^ (− 3.387)	−40.079^***^ (− 2.820)
South	−21.428^***^ (− 2.884)	−4.618 (− 0.720)	−66.988^***^ (− 6.268)	−65.236^***^ (−5.319)
Year = 2010	ref	ref	ref	ref
Year = 2011	2.035^**^ (2.281)	2.145^**^ (2.442)	− 11.747^***^ (− 3.391)	− 11.472^***^ (− 3.341)
Year = 2012	0.284 (0.245)	0.361 (0.313)	− 0.377 (− 0.131)	− 0.085 (− 0.030)
Year = 2013	2.124^*^ (1.658)	0.902 (0.642)	1.171 (0.297)	1.561 (0.409)
Year = 2014	4.162^***^ (2.770)	2.411 (1.444)	9.185^***^ (2.665)	9.536^***^ (2.736)
Year = 2015	6.655^***^ (4.041)	4.590^**^ (2.464)	12.958^***^ (3.546)	13.286^***^ (3.584)
Year = 2016	11.106^***^ (6.147)	8.750^***^ (4.314)	18.269^***^ (4.645)	18.556^***^ (4.656)
Year = 2017	13.444^***^ (6.501)	10.998^***^ (4.842)	31.563^***^ (7.529)	31.906^***^ (7.199)
Rural (non-core)		−17.050^***^ (−4.452)		− 13.390^**^ (− 2.208)
Most disadvantaged		−9.220 (− 1.388)		−34.217^***^ (− 2.603)
Second most disadvantaged		−9.301^**^ (−2.294)		−10.721 (− 1.229)
Third most disadvantaged		−4.110 (− 1.417)		−3.483 (− 0.451)
Conditional community benefit requirement		−15.714^***^ (−3.914)		4.512 (0.516)
Minimum community benefit requirement		14.309^***^ (3.360)		33.216^***^ (3.147)
Stricter community benefit plan		11.661^***^ (2.962)		8.409 (0.770)
No sales/use tax exemption		15.739^***^ (3.299)		9.435 (1.086)
Medicaid covers non-disabled adults		3.391^**^ (2.436)		−0.229 (− 0.073)
Parents income eligibility limit		0.038^***^ (2.651)		−0.008 (− 0.266)
Constant	187.810^***^ (22.574)	175.010^***^ (23.595)	358.001^***^ (31.240)	351.245^***^ (26.999)
Observations	16,269	16,269	16,378	16,378

*t* statistics in parentheses

^*^p < 0.1, ^**^p < 0.05, ^***^p < 0.01

On the other hand, a hospital in a joint venture (15.66%, *p* < 0.01), in a highly concentrated market (14.14%, *p* < 0.01), and in a state with a minimum community benefit requirement (33.22%, *p* < 0.01), was associated with a statistically significant higher family income eligibility limit for discounted care. The results also indicate that hospitals located in the Northeast, Midwest, and South regions generally offer less generous limits than hospitals in the West region. In terms of the year effects, the hospitals, all else being equal, offered statistically significant higher eligibility limits in 2017 as compared to 2010.

Table 3 shows the average marginal effects regression results investigating whether the hospital, community, and state policies are associated with the low, medium, or high family income eligibility limits. Both critical access and safety-net hospitals were associated with a high likelihood of offering low family income limit for free care (40–150% of FPL) and less likely to offer medium (200% of FPL) or high (160–600% of FPL). For example, safety-net hospitals are 10.1 (*p* < 0.01) percent more likely to offer low family income eligibility limit and 7.7 (*p* < 0.01) and 2.4 (*p* < 0.01) percent less likely to offer medium or high limit, respectively. In general, several hospitals’ community factors were associated with negative or positive statistically significant eligibility limits. A hospital in a joint venture or highly concentrated service area market was associated with higher eligibility limits. Also, state regulatory factors such as minimum community benefits requirements and stricter community benefits requirements were associated with more likelihood of offering medium and high-income eligibility for free and discounted care. Hospitals located in rural areas, located in most disadvantaged areas, are in states with conditional benefit requirements that were less likely to offer statistically significant medium or high eligibility levels. On average, hospitals were more likely to have increased eligibility limits in 2017 than in 2010.

**Table 3:** Average marginal effects of the probability of offering low, medium, or high family income eligibility category for free or discounted care

	Family income eligibility categories for free care.			Family income eligibility categories for discounted care.		
	Low (40–150% of FPL)	Medium (160–200% of FPL)	High (201–600% of FPL)	Low (0–300% of FPL)	Medium (301–400% of FPL)	High (401–800% of FPL)
Non-critical access hospital	ref	ref	ref	ref	ref	ref
Critical access hospital	0.091^**^ (2.188)	−0.070^**^ (−2.138)	− 0.022^**^ (− 2.008)	0.089^**^ (2.005)	−0.079^**^ (− 1.999)	−0.010^*^ (− 1.764)
Non-safety-net	ref	ref	ref	ref	ref	ref
Safety-net	0.101^***^ (3.403)	−0.077^***^ (−3.159)	− 0.024^***^ (−2.981)	0.055 (1.307)	−0.048 (−1.302)	− 0.006 (− 1.257)
Not-for-profit private	ref	ref	ref	ref	ref	ref
Public	0.078^*^ (1.796)	−0.060^*^ (−1.764)	− 0.019^*^ (− 1.705)	0.072 (1.521)	−0.064 (− 1.522)	−0.008 (− 1.388)
Not-for-profit, church	− 0.051 (− 1.465)	0.039 (1.430)	0.012 (1.470)	− 0.022 (− 0.513)	0.020 (0.511)	0.003 (0.524)
Small (< 100 beds)	ref	ref	ref	ref	ref	ref
Medium (100–399 beds)	−0.017 (− 0.422)	0.013 (0.423)	0.004 (0.415)	− 0.060^*^ (− 1.817)	0.053^*^ (1.817)	0.007 (1.613)
Large (400+ beds)	0.052 (1.043)	−0.039 (− 1.023)	− 0.012 (− 1.067)	−0.047 (− 0.916)	0.042 (0.922)	0.006 (0.845)
Non-teaching	ref	ref	ref	ref	ref	ref
Major teaching	−0.051 (−1.203)	0.039 (1.196)	0.012 (1.165)	−0.079^**^ (−1.971)	0.070^*^ (1.938)	0.009^*^ (1.904)
Minor teaching	0.041 (1.288)	−0.031 (−1.284)	− 0.010 (− 1.232)	0.028 (0.814)	− 0.025 (− 0.815)	−0.003 (− 0.792)
Not in a joint venture	ref	ref	ref	ref	ref	ref
In a joint venture	−0.071^***^ (−2.809)	0.054^***^ (2.697)	0.017^**^ (2.476)	−0.120^***^ (−5.124)	0.106^***^ (5.081)	0.014^***^ (2.920)
Stand-alone hospital	ref	ref	ref	ref	ref	ref
Health system hospital	−0.064 (−1.640)	0.049 (1.629)	0.015 (1.529)	−0.006 (− 0.167)	0.006 (0.167)	0.001 (0.167)
Lowly concentrated (< 0.33 on HHI)	ref	ref	ref	ref	ref	ref
Moderately concentrated (0.3387 to 0.8401 on HHI)	0.026 (1.237)	−0.020 (−1.213)	− 0.006 (− 1.251)	−0.031 (− 1.416)	0.027 (1.411)	0.004 (1.339)
High concentrated (0.8403 to 1 on HHI)	−0.065^**^ (− 1.962)	0.049^*^ (1.943)	0.016^*^ (1.785)	− 0.113^***^ (− 3.659)	0.100^***^ (3.567)	0.013^***^ (2.780)
Financial assistance share of total expense	−0.010 (− 1.539)	0.007 (1.522)	0.002 (1.470)	−0.010^*^ (− 1.773)	0.009^*^ (1.755)	0.001^*^ (1.689)
Urban	ref	ref	ref	ref	ref	ref
Rural (non-core)	0.172^***^ (4.283)	−0.131^***^ (−4.020)	− 0.041^***^ (−3.142)	0.131^**^ (2.551)	− 0.116^**^ (−2.546)	−0.015^**^ (− 2.069)
Most disadvantaged	0.085 (1.239)	−0.064 (−1.228)	− 0.020 (− 1.211)	0.314^***^ (2.949)	−0.278^***^ (− 2.899)	−0.037^**^ (− 2.434)
Second most disadvantaged	0.058 (1.079)	−0.044 (− 1.069)	−0.014 (− 1.067)	0.067 (0.910)	− 0.059 (− 0.910)	−0.008 (− 0.883)
Third most disadvantaged	0.058 (1.339)	−0.044 (− 1.324)	−0.014 (− 1.304)	−0.021 (− 0.395)	0.018 (0.394)	0.002 (0.394)
Conditional community benefit requirement	0.204^***^ (4.587)	−0.155^***^ (− 4.286)	−0.049^***^ (− 3.231)	0.062 (1.018)	−0.055 (− 1.016)	−0.007 (− 0.992)
Minimum community benefit requirement	− 0.131^***^ (− 2.823)	0.100^***^ (2.748)	0.031^**^ (2.399)	−0.276^***^ (− 3.972)	0.244^***^ (4.071)	0.032^**^ (2.421)
Stricter community benefit plan	− 0.127^***^ (− 3.045)	0.097^***^ (2.813)	0.030^***^ (2.893)	− 0.121^**^ (− 2.237)	0.107^**^ (2.253)	0.014^*^ (1.813)
No sales/use tax exemption	− 0.099^**^ (− 1.979)	0.075^*^ (1.943)	0.024^*^ (1.842)	− 0.061 (− 0.909)	0.054 (0.910)	0.007 (0.875)
Medicaid covers non-disabled adults	− 0.045^***^ (− 2.767)	0.034^***^ (2.621)	0.011^**^ (2.556)	0.004 (0.169)	− 0.003 (− 0.169)	−0.000 (− 0.170)
Parents income eligibility limit	− 0.001^**^ (− 2.574)	0.000^**^ (2.550)	0.000^**^ (2.171)	0.000 (0.391)	− 0.000 (− 0.391)	−0.000 (− 0.388)
Northeast	− 0.151^**^ (− 1.968)	0.114^*^ (1.912)	0.036^*^ (1.890)	0.323^***^ (3.681)	− 0.285^***^ (− 3.697)	−0.038^**^ (− 2.480)
Midwest	0.015 (0.197)	−0.011 (− 0.197)	−0.004 (− 0.196)	0.310^***^ (4.620)	−0.274^***^ (− 4.573)	−0.036^***^ (− 2.848)
South	− 0.056 (− 0.832)	0.043 (0.826)	0.013 (0.830)	0.413^***^ (6.061)	− 0.365^***^ (− 5.988)	−0.048^***^ (− 3.069)
Year = 2010	Ref	Ref	Ref	Ref	Ref	Ref
Year = 2011	−0.037^***^ (− 3.452)	0.031^***^ (3.262)	0.006^***^ (2.750)	0.037^**^ (2.365)	−0.034^**^ (− 2.383)	− 0.003^*^ (− 1.754)
Year = 2012	− 0.027^*^ (− 1.757)	0.023^*^ (1.709)	0.004^*^ (1.783)	− 0.028 (− 1.571)	0.026 (1.552)	0.002 (1.577)
Year = 2013	− 0.037^*^ (− 1.887)	0.031^*^ (1.821)	0.006^**^ (1.978)	−0.051^**^ (− 2.234)	0.047^**^ (2.186)	0.005^**^ (2.188)
Year = 2014	−0.065^***^ (− 2.645)	0.054^**^ (2.489)	0.011^***^ (2.726)	−0.085^***^ (− 3.235)	0.077^***^ (3.138)	0.008^***^ (2.702)
Year = 2015	−0.081^***^ (− 2.977)	0.066^***^ (2.787)	0.015^***^ (2.858)	−0.129^***^ (− 4.378)	0.116^***^ (4.170)	0.014^***^ (3.157)
Year = 2016	−0.126^***^ (− 4.276)	0.098^***^ (3.749)	0.029^***^ (3.621)	−0.165^***^ (− 4.691)	0.147^***^ (4.439)	0.019^***^ (3.299)
Year = 2017	−0.162^***^ (− 5.051)	0.118^***^ (4.160)	0.044^***^ (3.869)	−0.213^***^ (− 5.460)	0.186^***^ (5.143)	0.027^***^ (3.322)
Observations	16,269	16,269	16,269	16,378	16,378	16,378

*t* statistics in parentheses

^*^p < 0.1, ^**^p < 0.05, ^***^p < 0.01

## Discussion

This study’s purpose was two-fold; first, investigate whether hospital characteristics and community factors are associated with the eligibility limits. Second, the association, if any, of the state regulatory environment on eligibility limits for free and discounted care. The results suggest that overall, the variation in the family income eligibility limits for free and discounted care, to no small extent, might be explained by differences between the hospital service area nested in the hospital referral regions. This appears to be a good sign as one could expect the family income eligibility limits to mirror the local economic environment. In other words, hospital services areas with low average income might be expected to have low eligibility levels, while those in high-income areas might have a higher family income limit level. However, there are two areas the results indicate might be of concern. The results show that anywhere from 30 to 45% of variation might be explained by hospital service area variation. One can interpret that even after controlling for local economic factors and state regulatory factors, there is still variation that is not accounted for, and therefore not based on the local conditions.

Second, the results highlight that those hospitals that serve low-income and underserved communities are less able to or less likely to offer more relief in terms of free or discounted care. Hospitals such as critical care, safety-net, or public hospitals might be the only hospitals in the community. The results suggest the likelihood of two underlying factors that might be influencing the decision on free and discounted care generosity: financial capability and market concentration. There is evidence that safety-net, teaching, critical care, and rural hospitals often operate with thin operating margins; therefore, they can least afford to be generous in terms of high family income eligibility limits [29]. The results show that hospitals in joint-ventures and highly concentrated markets offer more generous eligibility limits. The findings might seem to bolster the arguments of mergers and general hospital consolidations. Hospitals with a considerable market share will enjoy economies of scale, the argument goes, have substantial negotiating power with payers and suppliers, and offer more generous community benefits. However, research has shown that hospital consolidation through mergers and acquisitions lead to an increase in prices, a modest decline in inpatient experiences, no measurable change in healthcare quality, and sometimes curtailing unprofitable services [30–34].

The second study question was to examine the association between state regulatory environment regarding the provision of community benefits by NFP hospitals and hospitals’ likelihood of providing higher family income eligibility limits. The study focused on four state regulations governing tax-exempt hospitals and one policy decision. These were: community benefit requirement, minimum community benefit requirement, stricter community benefits plan (implementation strategy) than the IRS requirement plan, and no exemption from sales and tax regulations. The regulations included whether a state has the provision of community benefit requirements. The community benefit requirement varies from state to state but might include providing benefits as a condition of any of the following: certificate of need approval, property tax exemption, hospital licensure, sales tax exemption, or partial state reimbursement for charity care expenses. The study also included a state policy decision on whether Medicaid covers non-disabled adults, essentially an indicator of Medicaid expansion.

Among the four regulation requirements and the Medicaid expansion policy, the minimum community benefit requirement appears to be associated consistently with higher family income eligibility limits for free care and discounted care, and the increased probability of offering high free and discounted care. Similarly, stricter community plan, no sales and use tax exemption, and coverage of non-disabled adults were also associated with increased eligibility limits and the likelihood of offering higher eligibility limit for free care, but not discounted care. Interestingly, conditional community benefits requirement was associated with lower family income eligibility limit and increased probability of offering a low eligibility limit for free care. The results suggest that states have options to consider nudging the tax-exempt hospitals to offer more relief to local communities. The minimum community benefit requirement appears to be the one regulation associated with an increase in the likelihood of offering high eligibility limits for free and discounted care. Overall, the results are in line with previous studies that found that states with stricter tax-exempt rules and those in states that expanded Medicaid seem to be more generous in offering comprehensive community benefits [35, 36].

It is expected that some hospitals are going to face a financial struggle in the coming months and even years following the disruptions as a result of the COVID-19 pandemic [37]. Federal and state-level policymakers might need to consider offering targeted relief with hospital assistance requirements in terms of free and discounted care as part of the community benefits package. Critical care, safety-net, and rural hospitals happen to be the ones least likely to offer generous free and discounted care limits and yet might also be serving minority communities that have been critically impacted by the pandemic. However, care should be taken to avoid unintended consequences including forcing them to close due to unsustainable financial obligation. Following the 2008–09 economic recession, and several years later, rural communities, for example, continue to see a rapid closure of hospitals. Therefore, offering relief to these hospitals will serve two-fold purposes, keep them open, and offer more community benefits for communities hungering for the benefits.

### Limitations

The issue of family income eligibility for care is complicated, and the reported limits do not mean hospitals do not have other ways of offering relief to the communities, including discounts on catastrophic health expenditures. Further, like other government data used for regulatory purposes, the IRS reports often need cleaning; therefore, a different cleaning approach might result in slightly different estimates.

### Future research directions

Affordability of health care is critical especially following the recent economic downturn, pandemic and demonstration for racial equity. While more research has previous focused on overall community benefits for NFP hospitals more is needed to understand how hospital policies such as financial assistance and collection policies affect patient and communities, and whether these policies exacerbate health inequities. Further, a more thorough investigation is needed to understand whether hospitals that offer less relief also charge less as compared to counterparts.

## Data Availability

The primary (IRS form 990) data that support the findings of this study are available from Applied Nonprofit Research, but restrictions apply to the availability of these data, which were used under license for the current study, and so are not publicly available.
